# Evaluating large language models in patient education: a comparative analysis addressing frequently asked questions in peri-acetabular osteotomy

**DOI:** 10.1308/rcsann.2026.0056

**Published:** 2026-08-27

**Authors:** TP Davis, B Guevel, K Logishetty, AG Dick, J Hutt

**Affiliations:** University College London Hospitals NHS Foundation Trust, UK

**Keywords:** Artificial intelligence, Generative AI, Large language models, Peri-acetabular osteotomy, Patient education

## Abstract

**Introduction:**

Large language models (LLMs) are increasingly used as sources of information across many fields, including healthcare. As patients turn to these models for health-related queries, evaluating the accuracy and reliability of their responses is essential. Peri-acetabular osteotomy (PAO) is performed on younger patients – a group more likely to use digital tools like LLMs for health information. This study assesses the accuracy and readability performance of two leading LLMs, ChatGPT and Google Gemini, in addressing common patient questions on PAO.

**Methods:**

A panel of fellowship-trained PAO surgeons created ten commonly asked patient questions based on real-world experience. Responses from each LLM were assessed by the same three surgeons, blinded to response origin, using a 5-point Likert scale to evaluate clarity, accuracy, and completeness. Readability was measured with Flesch–Kincaid Reading scores.

**Results:**

ChatGPT outperformed Gemini with an average score of 4.17 vs 3.13 (*t* = −3.08, *p* = 0.006). ChatGPT’s responses were often rated higher for completeness and clarity, particularly in areas needing detailed explanation, and usually required minimal clarification. Gemini sometimes lacked specificity or included minor inaccuracies that reduced its perceived reliability. Both LLMs produced responses with similar “difficult” Flesch Reading Ease scores.

**Conclusions:**

There may be significant differences in how effectively LLMs support patients with surgical queries. ChatGPT more consistently met expert standards for clarity and thoroughness. As LLM usage expands, ChatGPT may aid patient education on hip surgery, supporting consultations, informed decisions and postoperative guidance.

## Introduction

Large language models (LLMs) are artificial intelligence (AI) programs trained on extensive data sets to generate coherent text. Among the most widely recognised free open-access models are OpenAI’s ChatGPT and Google DeepMind’s Gemini.^1,2^ Recently, rapid advancements in LLM development have been observed, likely driven by industry competition and their broad applicability, including in healthcare.^3^

Peri-acetabular osteotomy (PAO) surgery is established as an effective intervention for the treatment of hip dysplasia in appropriately selected patients.^4,5^ Originally described by Ganz *et al* in 1988, the procedure has gained popularity as a joint-preserving surgery; it is particularly suited to younger patients with hip dysplasia, without significant degenerative change, who have not achieved adequate symptom relief with conservative measures.^6,7^ In addition to hip dysplasia, PAO is also indicated for conditions such as hip impingement due to acetabular retroversion.^8,9^

These conditions generally affect a younger population who are increasingly exposed to AI and smart-assistant technologies, which are now a regular part of their daily lives and workplaces.^10^ As a result, they may be more likely to use LLMs to research potential treatments for their medical problems, such as PAO surgery. The search for relevant information may occur both before formal clinical evaluation or in the period of preparation for decision making or surgery itself.

Previous research has examined ChatGPT’s responses within the context of delivering patient information.^11,12^ However, to date, a direct comparative analysis of responses from ChatGPT and Gemini has not been conducted in the context of patient-focused frequently asked questions (FAQs) for PAO surgery. Although similar comparative studies have been performed for other health conditions, this gap highlights the need for a focused evaluation of these LLMs in addressing questions commonly posed by patients undergoing orthopaedic care.^13–15^ PAO surgery is a good example of a complex intervention performed in a younger age group who may be more inclined to use up-to-date technology in their own exploration of their condition and treatment options. The aims of this study were to evaluate the accuracy and readability of responses provided by ChatGPT and Gemini, and to compare the performance of these LLMs in delivering patient-focused information in this specific clinical context.

## Methods

An expert panel of three experienced, fellowship-trained PAO surgeons curated ten FAQs regarding PAO surgery, commonly encountered in the outpatient setting. These questions were phrased to simulate queries a patient might input into publicly available LLMs, without any specific prompts provided to guide the responses.

These questions were entered into the open-access, unlogged-in versions of ChatGPT-4 and Google Gemini after deletion of cookie and location data from the browsers. The responses were then independently assessed by all three surgeons using a modified 5-point Likert scale, with any discrepancies in scores discussed and resolved through consensus. The Likert scale used for evaluation was as follows:

- 5 points: The response is completely accurate, clear and thorough, directly addressing the question without factual errors and providing all necessary details.
- 4 points: The response is mostly accurate and clear but may omit minor details or contain slight ambiguities. It adequately answers the question.
- 3 points: The response is generally accurate but contains some omissions or minor factual errors. It addresses the question but lacks depth in certain areas.
- 2 points: The response contains notable inaccuracies, lacks clarity or is only partially relevant to the question. Significant improvements are required.
- 1 point: The response is largely inaccurate, unclear or irrelevant, failing to provide useful or correct information.

The reviewing surgeons were also given the option to provide comments on each response, which were later analysed separately from the quantitative data. To minimise bias, reviewers were blinded to the source of each response during the evaluation process. The ratings provided by each reviewer were analysed, and the performance of the two LLMs was compared quantitatively based on their overall scores. The LLM answers were also assessed for readability using Flesch Reading Ease and Flesch–Kincade Grade Level scores.

Continuous data, including Likert scores, were summarised using median and interquartile range (IQR) to account for their ordinal nature. Normality of data distribution was assessed using the Shapiro–Wilk test. Chat GPT Likert scores (*p* = 0.0006) and Flesch Reading Ease score differences (*p* = 0.0157) were not normally distributed and so a Wilcoxon signed rank test was used for paired comparisons. A paired *t* test was used for the Flesch–Kincade Grade Level scores, because the differences were normally distributed (*p* = 0.3869). Potential sources of bias, such as variability across reviewers, were minimised by using paired analyses to compare scores for the same questions across models. A *p*-value of <0.05 was considered statistically significant. All analyses were performed using R (v.4.3.2, R Core Team 2021), with visualisations created using the ggplot2 package.

## Results

### Accuracy

Ten responses from each LLM were received to the questions in Table 1. ChatGPT’s responses were rated significantly higher for accuracy (median Likert score: 4.0; IQR: 1) than Gemini’s responses (median: 3.0; IQR: 1) (*p* < 0.01) (Figure 1 and Table 1). The mean Likert grade for ChatGPT’s responses was 4.07, whereas for Gemini it was 3.23. ChatGPT received 13 scores of 5 compared with 3 for Gemini. Some 86.7% of ChatGPT’s responses were scored ≥4 for accuracy, for Gemini this was 30%.

**Figure 1:**
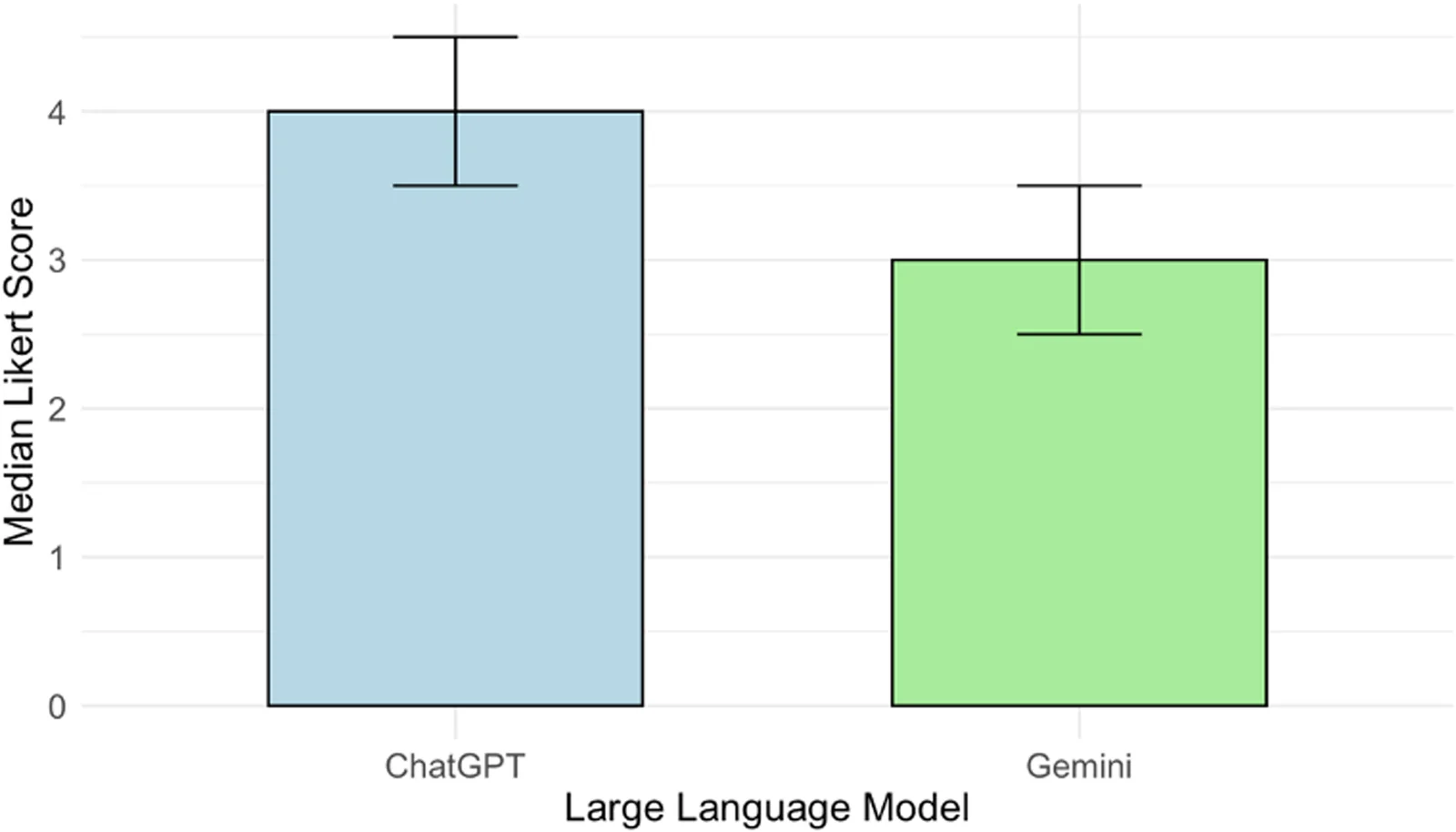
Comparison of median score between Gemini and ChatGPT

**Table 1:** Questions asked, scores allocated and abbreviated answers from the large language models

		Average Likert score		Flesch Reading Ease score		Word count	
No.	Question	ChatGPT	Gemini	ChatGPT	Gemini	ChatGPT	Gemini
1	What is peri-acetabular osteotomy (PAO) surgery?	4 (4–5)	3 (3–4)	50.6	39.5	355	207
2	How does PAO surgery help in treating hip dysplasia?	5 (4–5)	3 (3–4)	40.3	30	588	145
3	What are the risks and complications associated with PAO surgery?	4 (2–4)	2 (2–3)	33.7	39.3	918	195
4	How long does PAO surgery take?	4 (4–5)	3 (3–3)	27.2	32.9	123	26
5	What is the expected recovery time after PAO surgery?	5 (3–5)	3 (3–4)	31.1	31.2	563	132
6	Will I need physical therapy after the surgery?	5 (4–5)	4 (3–5)	29.8	31.8	632	99
7	How much pain should I expect post-surgery and how is it managed?	5 (4–5)	4 (3–5)	41.2	32.9	1064	143
8	What are the success rates of PAO surgery?	4 (4–5)	3 (3–5)	40.4	30.5	653	139
9	Are there any alternatives to PAO surgery for treating hip dysplasia?	2 (1–4)	2 (1–2)	30.9	59.2	1021	169
10	How should I care for the surgical incision site?	5 (4–5)	3 (1–4)	51.9	41.4	940	169
Average score		4.07	3.23	37.71	36.97	685.7	142.4

### Readability

The average Flesch Reading Ease scores were 36.87 for Gemini and 37.71 for ChatGPT, both indicating a “difficult” readability level, with no significant difference between the two (*p* = 0.4922). The average Flesch–Kincaid Grade Level scores were 11.82 for Gemini and 11.32 for Chat GPT, corresponding to an 11th–12th grade reading level, with no statistically significant difference (*p* = 0.5157) (Table 2). An example of the LLMs’ answers to one question is demonstrated in full in Table 3, showing ChatGPT’s answer of 355 words and Gemini’s of 207 words. ChatGPT provided answers that were on average 79.2% longer than Gemini’s responses, corresponding to an average length discrepancy of 543 words. Full-text answers to all questions are in given in Appendix 1 (available online).

**Table 2:** Readability metrics

Metric	Gemini	ChatGPT	*p*-value
Flesch Reading Ease score	36.87	37.71	0.4922
Flesch–Kincaid Grade level	11.82	11.32	0.5157

**Table 3:** Example of large language model answers

	Answer	
Question	Gemini	ChatGPT
What is peri-acetabular osteotomy (PAO) surgery?	Peri-acetabular osteotomy (PAO) is a surgical procedure used to treat hip dysplasia, a condition where the hip socket (acetabulum) is too shallow or improperly shaped. This misalignment can lead to pain, stiffness, and early arthritis.**How does PAO work?** **Repositioning the acetabulum:** The surgeon makes a series of cuts around the acetabulum, allowing it to be repositioned and realigned. **Fixing the new position:** Screws or metal plates are used to secure the acetabulum in its new position. **Improved hip function:** By creating a better fit between the hip ball and socket, PAO can alleviate pain, improve range of motion, and delay the onset of arthritis. **Who is a candidate for PAO?** Typically, individuals who are diagnosed with hip dysplasia at a young age and are experiencing pain or limited mobility may be considered for PAO. The surgery is often performed before the development of severe arthritis.**Recovery from PAO:** Recovery from PAO surgery can take several months. Patients typically require physical therapy to regain strength and mobility. The long-term success of the procedure depends on factors such as the severity of the hip dysplasia and the patient's adherence to postoperative care.**Would you like to know more about the procedure, recovery process, or potential risks and benefits?**	Peri-acetabular osteotomy (PAO) is a surgical procedure used to treat hip dysplasia, a condition in which the acetabulum (the socket of the hip joint) does not cover the head of the femur (the ball of the hip joint) adequately. This can lead to instability, pain, and the early development of osteoarthritis.**Key aspects of PAO surgery:** 1. **Goal**: The primary goal of PAO surgery is to improve the coverage of the femoral headby repositioning the acetabulum. This enhances joint stability, reduces pain, and can delay or prevent the need for a hip replacement. 2. Procedure: The surgeon makes an incision around the hip area. Several cuts (osteotomies) are made in the pelvic bone near the acetabulum. The acetabulum is repositioned to better cover the femoral head, improving the congruency of the hip joint. The bones are then fixed in the new position with screws or plates to allow them to heal in place. 3. Indications: PAO is typically recommended for young to middle-aged adults who have symptomatic hip dysplasia and no significant hip joint damage (cartilage is still relatively preserved). It is often used when non-surgical treatments, like physical therapy or pain management, are not effective in relieving symptoms. 4. Recovery: Recovery from PAO surgery can take several months. Patients typically require crutches for several weeks, followed by physical therapy to regain strength and mobility. Full recovery may take 6 to 12 months, but it varies depending on the patient’s condition, healing process and rehabilitation. 5. Outcomes: When performed early, before significant joint degeneration, PAO can relieve pain, improve function and delay the need for more invasive procedures like hip replacement. It is considered one of the more effective surgeries for preserving the hip joint in cases of dysplasia, particularly for young adults. 6. Risks: As with any major surgery, there are risks, including infection, nerve damage, blood clots and complications related to bone healing.PAO is considered a joint-preserving surgery, meaning that it is designed to save the natural hip joint and delay or avoid the need for a total hip replacement.

## Discussion

AI has been previously applied in many areas within the realm of hip preservation surgery, such as in predicting the likelihood of total hip arthroplasty (THA) following hip arthroscopy, developing three-dimensional hip models to assist PAO surgical planning and quantifying intraoperative cuts in PAO surgery; to the best of our knowledge no study has thus far evaluated the accuracy of LLM-generated responses to preoperative patient inquiries.^16–18^ Evidence suggests that AI may facilitate shared decision making, improve clinical efficiency, and enhance patient and clinician satisfaction during AI-assisted consultations.^19–21^ In this study, ChatGPT produced responses that were, on average, more accurate than those of Gemini with a greater number of “completely accurate” responses recorded for ChatGPT compared to Gemini.

One possible contributor to ChatGPT’s higher accuracy scores is its significantly longer responses – on average, 543 words more – which may have conveyed greater perceived detail. Reviewers also noted qualitative differences between the LLMs. ChatGPT generally provided more-detailed answers than Gemini, which were consistent with the existing literature. For example, ChatGPT mentioned that PAO can address acetabular retroversion (anteverting PAO), a procedure shown to yield sustained clinical improvements up to 11 years postoperatively, whereas Gemini omitted this.^22^ ChatGPT also included specific information on recovery, length of stay (2–5 days), and physical therapy timelines, aligning with published data.^23–25^ By contrast, Gemini’s response on hospital stay was vague, noting variability without elaboration. ChatGPT described a structured, evidence-based physical therapy progression beginning on postoperative day 1, continuing through discharge and potentially intensifying at 4–6 weeks for at least two months.^23–26,28^

There were instances in which ChatGPT lacked accuracy, even producing factual errors; one such instance being a statement of reduced risk of labral tear post-PAO surgery, although there is little evidence to support this. Outcomes in terms of progression of osteoarthritis, re-operation and pain in the short-term post-PAO surgery are equivalent between those patients diagnosed with and those without labral tears pre-PAO surgery.^29^ There were also instances when ChatGPT provided answers that were very prescriptive, with the detail provided opening the answer to debate and disagreement.

Both LLMs identified avascular necrosis (AVN) as a risk of PAO surgery, despite limited evidence suggesting increased risk of AVN or dislocation post-PAO. In fact, PAO combined with other techniques may reduce femoral head AVN in patients with Legg–Calvé–Perthes disease.^30,31^ Both models also provided inaccurate information regarding PAO success rates: Gemini offered a brief, incomplete list of poor outcome risk factors, whereas ChatGPT overstated 20-year success rates in a highly detailed response. Although the literature reports varying outcomes, a 2021 meta-analysis by Tan *et al* estimated a mean survival of 10 years, with improved function up to 11 years postoperatively.^22^ In addition, both LLMs suggested inaccurate surgical alternatives. Gemini recommended hip arthroscopy for mild-to-moderate dysplasia, although evidence supports its use only in cases of borderline dysplasia.^32,33^ ChatGPT’s detailed response included hip resurfacing, shelf osteotomy and platelet-rich plasma (PRP) injections. Hip resurfacing, a bone-preserving form of arthroplasty for young patients with osteoarthritis, does not reorient the acetabulum; shelf osteotomy is rarely used today, although some advocate for its selective application; and PRP injections have only shown benefit in animal studies.^34–36^

Another area highlighted as needing improvement was the LLMs’ use of American terminology, such as the drug name “acetaminophen”, despite the LLMs generating responses to questions asked from an IP address located in the United Kingdom. This could confuse patients pre-PAO surgery – some may not be familiar with these terms. More importantly, concerns may be raised and confusion may occur when asking LLMs questions in other languages because the nuances of translations may be missed when generating responses if there is a bias towards answering as if the patient is based in the United States.^37^

Prior studies have evaluated the accuracy of LLMs in answering orthopaedic patient FAQs, with some comparing different LLMs or contrasting LLM and surgeon responses from the patient perspective.^11,21,38–43^ However, no studies have assessed LLM accuracy specifically for FAQs prior to PAO surgery, or compared Gemini and ChatGPT in this context. ChatGPT has previously provided appropriate responses to post-THA patient FAQs when compared with arthroplasty nurses, although further validation was recommended.^21^ Mika *et al* found an earlier ChatGPT version offered generally accurate pre-THA information, with 70% of responses requiring little to no clarification, and concluded that the content was accessible and understandable to patients.^11^ In this study, both LLMs produced more accurate responses than those reported by Mika *et al*, although higher readability scores suggest increased complexity of answer in newer versions.

A previous study found that the ChatGPT version assessed in our research provided acceptable and complete responses to shoulder pathology questions, with experts rating 25 answers as “nearly all correct”.^38^ Our findings indicate similar accuracy for ChatGPT, although Gemini performed slightly less accurately. By contrast, another study comparing LLM responses to rotator cuff tear FAQs reported equivalent median Likert scores for both ChatGPT and Gemini (5 out of 5).^42^ The reason for differing accuracy in responses to PAO-related FAQs remains unclear. Owing to the nature of LLM training on vast data sets, often derived from unverified online sources, LLMs may generate responses that are “untruthful” or “hallucinatory”.^44^ This underscores the importance of caution when considering their use in patient education. To avoid the presentation of incorrect information to patients, LLMs could be created by healthcare professionals, trained on verified healthcare data sets – similar to work carried out in 2023 that saw researchers develop a healthcare-specific generative AI trained on billions of data sets from University of Florida Health clinical departments, based on the architecture of ChatGPT-3.^45^

The Flesch Reading Ease and Flesch–Kincaid Grade Level calculator findings align with prior studies that examined the readability and grade level of LLM-generated responses to patient FAQs, which also identified a “difficult” readability and advanced grade level.^46^ Notably, in other contexts, ChatGPT’s responses to patient FAQs have previously been reported to score as low as 22.6 on the Flesch Reading Ease scale, potentially indicating an improvement in the readability of ChatGPT’s outputs since that study was conducted.^47^ These findings suggest that unfiltered responses from LLMs may be more appropriate for individuals with high health literacy, particularly when combined with expert consultation, owing to the high reading grade required to fully comprehend the LLMs’ answers. The “difficult” readability scores indicate that those from a lower socio-economic background may need extra support from clinicians when using LLMs to help assist in their healthcare decisions because of reported associated lower reading grades in this cohort.^48,49^

It is likely that patients will increasingly turn to AI tools to research medical procedures, necessitating that healthcare providers offer accurate guidance or corrections as required. Notably, both ChatGPT and Gemini frequently advised consultation with a surgeon for further information, although this recommendation was not uniformly present in their responses.

Given the rapid advancement of AI technology, these results may quickly become outdated. The ratings by expert reviewers, although informed by substantial clinical experience, training and familiarity with current literature, remain subjective. The combined Likert system (accuracy, clarity, completeness) used by the research team may have obscured important distinctions. Subgroup analysis would be beneficial in future research – unfortunately, it was not within the scope of this work. The questions asked of the LLMs may not be an accurate representation of the most common questions asked by patients pre-PAO surgery; they are a subjective impression of the most commonly asked patient questions derived from the three expert surgeons’ experiences clinically. Future research should involve all stakeholders when developing questions and answers to commonly asked questions – including patients, clinicians and allied health professionals.

## Conclusion

This study’s findings suggest that LLMs have potential as adjuncts in patient education and shared decision making for pre-PAO surgical candidates, although the current unfiltered readability of their answers may pose a difficulty for some patients. The assessed version of ChatGPT provided more accurate answers than Gemini to patient FAQs prior to PAO surgery, although both produced factual errors. The difference in the readability of answers between the two LLMs was not significant. Further research and development should prioritise LLMs trained on verified healthcare-specific data sets to better support orthopaedic surgeons in preoperative education and shared decision making. Our study suggests that the technology is still too error-prone to recommend their general use in preoperative patient counselling

## Competing interests

The author/s declare no competing interests.

## Funding

The author/s received no financial support for the research, authorship and/or publication of this article.

## Ethics approval and consent to participate

Not required.

## Author contributions

**TP Davis:** Conceptualization, Data curation, Formal analysis, Investigation, Methodology, Project administration, Resources, Software, Supervision, Validation, Visualization, Writing – original draft, Writing – review & editing. **B Guevel:** Conceptualization, Data curation, Formal analysis, Investigation, Methodology, Project administration, Resources, Software, Supervision, Validation, Visualization, Writing – original draft, Writing – review & editing. **K Logishetty:** Conceptualization, Formal analysis, Investigation, Methodology, Project administration, Supervision, Visualization, Writing – review & editing. **AG Dick:** Conceptualization, Formal analysis, Investigation, Methodology, Supervision, Visualization, Writing – review & editing. **J Hutt:** Conceptualization, Formal analysis, Investigation, Methodology, Project administration, Supervision, Visualization, Writing – original draft, Writing – review & editing.

## Artificial Intelligence

The author/s declare that AI (ChatGPT) was used to help improve the grammar, clarity and readability of this manuscript. After every use of the tool, the authors reviewed, edited and verified the content to ensure it accurately represents their own ideas and they take full responsibility for the final text.

## Data availability

All study data can be made available on request allowing for a 28-day response time.

## Supplementary Information

The online version contains supplementary material available at https://doi.org/10.1308/rcsann.2026.0056.
